# Agreement between self-reported and objectively measured hypertension diagnosis and control: evidence from a nationally representative sample of community-dwelling middle‐aged and older adults in China

**DOI:** 10.1186/s13690-024-01456-5

**Published:** 2024-12-26

**Authors:** Jingxian Wu, Danlei Chen, Cong Li, Yingwen Wang

**Affiliations:** School of Economics and Finance, Xi’an Jiaotong University, No 74 West Yanta Road, Yanta District, Xi’an, Shaanxi 710061 PR China

**Keywords:** Self-report, Objective measurement, Hypertension diagnosis, Hypertension control, Middle-aged and older Chinese adults

## Abstract

**Background:**

As the population ages, hypertension has become the leading risk factor for cardiovascular diseases (CVDs) and premature deaths worldwide. Accurate monitoring of CVD risks and planning community-based public health interventions require reliable estimates of hypertension prevalence and management. While the validity of self-reporting in assessing hypertension prevalence has been debated, the concordance between self-reports and clinical measurements of hypertension control remains underexplored, particularly in large, community-based older populations. This study aims to examine the agreement between self-reported and objectively measured data on both hypertension diagnosis and control, as well as the associated factors, among community-dwelling middle-aged and older Chinese adults.

**Methods:**

Data from the China Health and Retirement Longitudinal Study were utilized, with household survey responses combined with biomedical data. Sensitivity, specificity, and kappa coefficients were used to assess the agreement between self-reported and objectively measured hypertension diagnosis in the general sample, and the agreement on hypertension control among individuals who reported having hypertension. Binary and multinomial logistic regression analyses were conducted to identify individual, household, and community-level factors associated with the agreement.

**Results:**

Self-reports exhibited substantial sensitivity, excellent specificity, and moderate agreement with objective measurements for hypertension diagnosis, while demonstrating fair sensitivity, excellent specificity, but low agreement for hypertension control. The odds of agreement on hypertension diagnosis were negatively associated with older age and heavy drinking, but positively related to marital status, higher education, chronic kidney disease, recent healthcare service utilization, and higher household economic levels. Meanwhile, the likelihood of agreement on hypertension control was negatively associated with older age, comorbid diabetes or cardiovascular disease, heavy drinking, BMI over 25, and antihypertensive medication adherence, but positively associated with recently healthcare service utilization.

**Conclusions:**

Self-reporting underestimated hypertension prevalence but significantly overestimated the hypertension control rates. For middle-aged and older Chinese adults, individual-level factors including age, multimorbidity, behavioural risks, and healthcare-seeking behaviours were identified as significant predictors of agreement between self-reported and objectively measured hypertension data. Recognizing these factors is essential for improving the accuracy of chronic condition estimates and facilitating targeted chronic disease management programs for China’s aging population and other developing countries with similar demographic and health challenges.

**Supplementary Information:**

The online version contains supplementary material available at 10.1186/s13690-024-01456-5.

**Table:** 

Text box 1. Contributions to the literature
• Despite ample evidence on the validity of self-reporting in assessing hypertension prevalence, the concordance between self-reports and clinical measurements of hypertension control remains underexplored, particularly in large, community-based older populations.
• This study revealed moderate agreement for hypertension diagnosis but low agreement for hypertension control among community-dwelling middle-aged and older Chinese adults, highlighting the need to integrate self-reported data with biometric examinations for accurate chronic disease burden estimates.
• Individual-level factors such as age, multimorbidity, behavioural risks, and healthcare-seeking behaviours were identified as significant predictors of the agreement, guiding targeted community-based public health interventions for aging populations.

## Background

With the global population aging, non-communicable diseases (NCDs) have become a critical public health issue. Hypertension emerges as the primary risk factor for cardiovascular diseases (CVDs) and premature mortality worldwide [1]. In 2019, over 1.28 billion adults aged 30 to 79 were living with hypertension, representing more than 40% of the global population and contributing to over 10.8 million deaths (19.2%) [1, 2]. The growing incidence of hypertension poses significant challenges for primary health care, particularly in low- and middle-income countries [3]. In China, where the prevalence of hypertension has been steadily increasing, it is estimated that over one-fourth of the adult population suffers from this condition, with a higher incidence observed among middle-aged and older adults [2, 4]. In response to the health challenges posed by hypertension, the Chinese government has prioritized the prevention and management of NCDs within its primary healthcare framework since the implementation of the New Healthcare Reform in 2009 [5]. The Healthy China 2030 plan, launched in 2016, further highlights this focus by advocating for community-based NCD prevention, early detection, and management strategies. Health management programs for chronic conditions, particularly high blood pressure (BP), have been integrated into the coverage of essential public health services funded by the government, aiming to delay disease progression and enhance quality of life for affected community-dwelling middle-aged and older adults [6, 7].

Accurate monitoring of CVD risks and the effectiveness of community-based public health interventions critically depend on reliable estimates of hypertension prevalence and management [8, 9]. The high costs and low efficiency associated with biomedical data collection, compounded by an incomplete family doctor system in developing countries like China [10], have led healthcare researchers and policy-makers to heavily rely on self-reported hypertension for estimating disease prevalence and burden in the population. These self-reported data are typically collected during voluntary healthcare facility visits or large-scale questionnaire surveys. However, their reliability has been increasingly questioned due to potential reporting errors, recall bias, and individuals’ reluctance to disclose their health conditions [11–13]. Many previous studies have indicated that these inaccuracies in self-reported data result in erroneous prevalence estimates. This not only hinders effective hypertension management and control [14, 15] but also complicates the empirical analysis of the relationships between socioeconomic status and chronic health conditions [16].

To enhance the accuracy of hypertension estimates, researchers are increasingly integrating questionnaire-based survey data with health examinations to explore the concordance between self-reported and clinically measured hypertension prevalence [17]. These studies validate self-reported hypertension diagnosis against biomedical measurements, often regarded as the “*gold standard*,” revealing significant discrepancies between the two [16, 18–21]. Furthermore, they indicate that the agreement between self-reported and clinically measured hypertension diagnoses varied by factors such as age, sex, education, economic status, and assessment methods [16–21]. Recent research even utilizes data from sources like the WHO-SAGE survey to conduct international comparisons on these discrepancies [22]. Behavioural risks, including alcohol consumption, smoking, and obesity, along with healthcare service utilization [23, 24] have been identified as predictors of undiagnosed or inaccurately reported hypertension.

Although extensive research has explored the agreement between self-reported and objective data regarding hypertension diagnosis or prevalence, less is known about the accuracy of self-reported BP control among diagnosed hypertensive individuals. Few studies have solely compared self-reported BP values with those obtained from home monitors or clinical measurements, revealing significant inconsistency [25–28]. Only one Canadian study has assessed the validity of self-reported BP control among patients in a primary care setting [29]. However, due to the cost and time-consuming nature of clinically measurements, these studies have primarily relied on relatively small samples and/or have been restricted to specific subgroups [21, 28], limiting the generalizability of the results. This highlights a significant research gap in understanding how accurately individuals with hypertension can report their BP control, particularly in large, community-based older populations. Meanwhile, there is limited knowledge regarding the variations and factors associated with the agreement on hypertension control between self-reports and biomedical measurements.

In this cross-sectional study, our aim was to thoroughly examine the agreement between self-reported and objectively measured hypertensive conditions among community-dwelling middle-aged and older Chinese adults, in response to the nation’s growing burden of NCDs associated with population aging. Utilizing data from the China Health and Retirement Longitudinal Study (CHARLS), we integrated self-reported questionnaire data with biometric health examinations to assess the concordance not only on hypertension diagnosis in the general sample but also on hypertension control among individuals who reported having hypertension. This approach enables a comprehensive evaluation of the accuracy of self-reported hypertension data across a nationally representative, community-wide older population. Furthermore, we investigated associated factors at the individual, household, and community levels with the agreement between self-reported and measured hypertension data, with the goal of identifying key factors related to the agreement. The results of our study provide crucial evidence to inform the implementation of targeted chronic disease management programs for older populations in China and other developing countries facing similar demographic and health challenges.

## Methods

### Study design

Based on descriptive statistical analysis, we employed a quantitative correlational study design to perform a secondary analysis of cross-sectional data from the CHARLS to clarify the associated factors with the agreement between self-reported and objectively measured hypertensive conditions among middle-aged and older Chinese adults. The reporting of this study adhered to the STrengthening the Reporting of Observational Studies in Epidemiology (STROBE) guidelines [30] (see Additional File 1 for the STROBE checklist).

### Data sources

Data for this study were derived from the CHARLS, a nationally representative survey conducted by Peking University focusing on community-dwelling middle-aged and older adults in China. The CHARLS survey utilized a multistage stratified probability sampling technique to obtain a representative sample of individuals aged 45 and older from 450 villages/communities across 28 provinces in China. The national baseline of the CHARLS survey took place in 2011–2012, with subsequent waves in 2013, 2015, and 2018. A structured questionnaire for face-to-face interviews served as the primary tool of the CHARLS survey, gathering data on respondents’ demographic backgrounds, health status and functioning, health care and insurance, occupational status, and household characteristics. To address potential errors in self-reported health variables, CHARLS interviewers also collected a series of biomarkers, including BP, for each respondent. This biomarker data was initially collected in the baseline wave (2011) and most recently updated in 2015. This study specifically analysed data from the 2015 wave, which included the most recent biomedical measurements and corresponding household questionnaire data, allowing for a comprehensive analysis of the concordance between self-reported and clinically measured hypertensive conditions. The CHARLS was approved by the Ethical Review Committee of Peking University (IRB 00001052–11014 & IRB00001052-11015), and informed consent was obtained from all participants. Further details on sampling procedure, data quality management, and biomarker process of the CHARLS can be found elsewhere [31, 32].

Data on self-reported hypertension diagnosis and control was collected through the household questionnaire segment of the CHARLS survey. Respondents were initially asked whether they had ever been diagnosed with hypertension by a healthcare professional, with response options of “no” or “yes.” Those who reported a hypertension diagnosis were then questioned about their BP control status, answering whether their BP was generally “under control” or not. Meanwhile, biomedical BP measurements were obtained by taking three readings at five-minute intervals on a single occasion, using an Omron model HEM-7112 electronic monitor during the physical examinations conducted by the CHARLS team. Consistent with previous research [20, 33], we used the average of these three measurements to determine each respondent’s actual BP values. Respondents who did not undergo the physical examination or had only one or two BP readings were excluded from the final analysis.

**Fig. 1:**
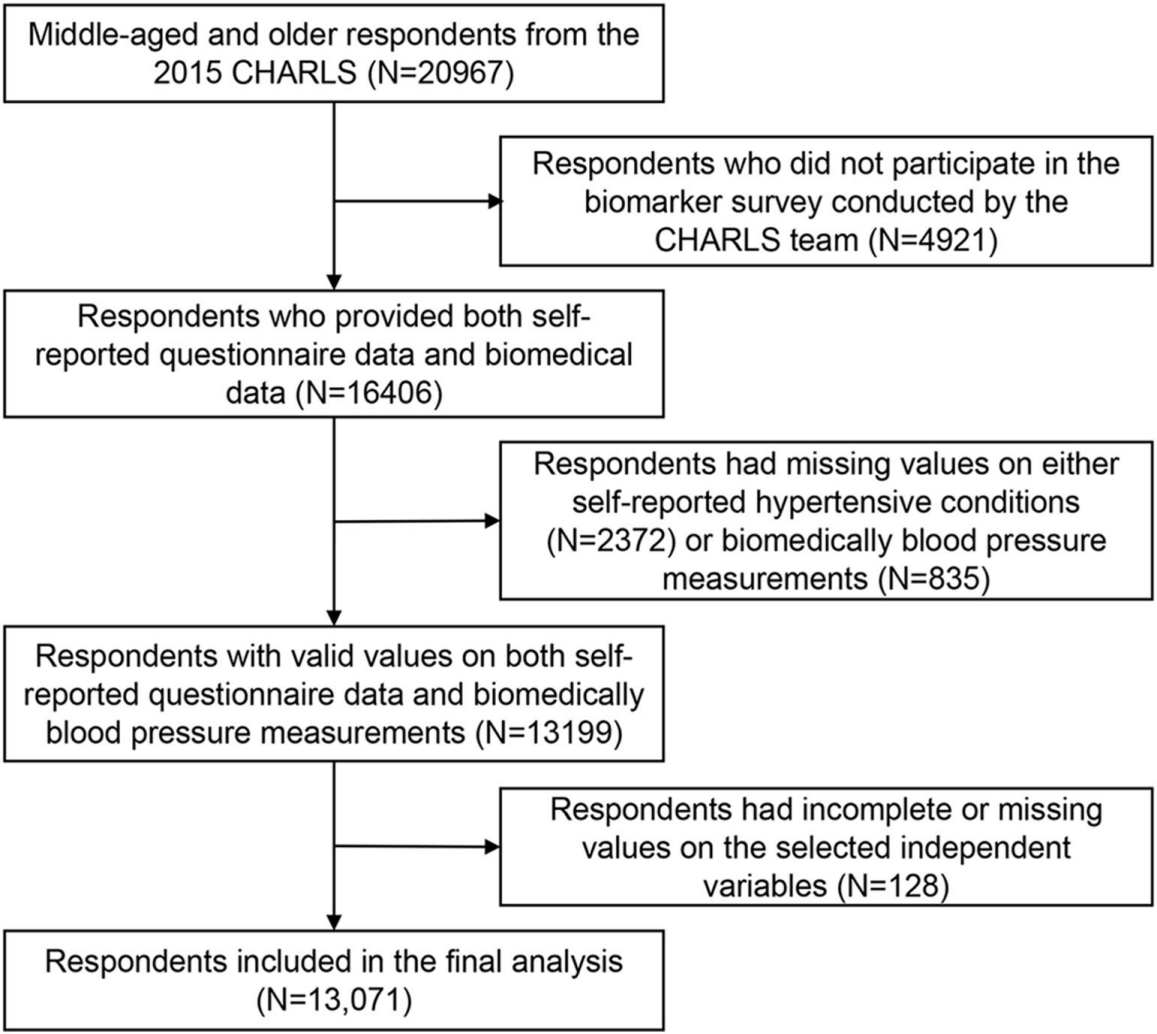
Sample inclusion process

From the 2015 CHARLS dataset, which initially included 20,967 respondents, 16,406 participated in the biomarker component. Our analysis was restricted to participants with valid data on both self-reported hypertensive conditions and biomedical BP measurements, reducing the sample size to 13,199 respondents. An additional 128 cases with incomplete or missing data on independent variables (as defined in the following section) were excluded. After these data cleaning procedures (as illustrated in Fig. 1), a final sample of 13,071 respondents remained for the following analysis.

## Measurements

### Dependent variable

The dependent variable in this study was the agreement between self-reported and objectively measured hypertensive conditions, evaluated in two ways: agreement on hypertension diagnosis for the general sample and agreement on hypertension control among those who reported having hypertension.

According to the World Health Organization (WHO) guidelines, biomedical hypertension was defined as having a systolic BP of 140 mm Hg or higher, a diastolic BP of 90 mm Hg or higher, and/or being on antihypertensive medication for the general population [1, 33]. For individuals with diabetes, CVDs (such as heart disease, stroke, and dyslipidaemia), or chronic kidney disease (CKD), hypertension was defined as a BP of 130/80 mm Hg or higher [1]. Control of hypertension was defined as maintaining an averaged BP below 130/80 mm Hg for those with the aforementioned comorbid conditions, or below 140/90 mm Hg for other hypertensive patients.

The concordance between self-reported and clinically measured hypertensive conditions was assessed using the categories True Positive (TP), True Negative (TN), False Positive (FP), and False Negative (FN). We created a dummy variable to measure the overall agreement, where TP and/or TN = 1 indicated agreement (yes = 1), and FP and/or FN = 1 indicated disagreement (no = 0). TP was defined as a correct match between self-reported and objectively measured hypertension diagnosis (or uncontrolled high BP). TN was assigned when respondents who self-reported no hypertension diagnoses (or controlled hypertension) were confirmed to have controlled BP based on biomedical data. FP occurred when respondents self-reported a hypertension diagnosis (or uncontrolled BP) without biomedical confirmation, while FN was recoded when respondents self-reported no hypertension diagnosis (or controlled high BP) but biomedical measurements indicated they were hypertensive (or had uncontrolled BP). Additionally, the two categories of false reporting (i.e., FP and FN) were analysed separately as outcome variables (yes = 1, no = 0) to explore the sources of overall discrepancies between self-reported and clinical measurements.

### Independent variables

Given the multistage sampling design of the CHARLS, this study employed various variables at the individual, household, and community-levels to characterize the sample and explore potential factors associated with the agreement between self-reported and objectively measured hypertension diagnosis and control.

Individual-level variables measured respondents’ demographic and socioeconomic characteristics, multimorbidity, behavioural risks, and healthcare-seeking behaviours [19, 20, 34]. Demographic and socioeconomic factors included age categorized into WHO-defined groups (middle-aged: 45–59 years; young-old: 60–74 years; old-old: 75 years and older), sex (female vs. male), marital status (unmarried vs. married), educational level (illiteracy; primary school; secondary school and above), and current occupational status (unemployed or retired; agricultural job; non-agricultural job). Multimorbidity was assessed by the self-reported presence of diabetes, CVD, and CKD (without vs. with). Behavioural risks included smoking status (never, former, or current smoker), heavy drinking status (consuming alcohol four or more days per week, no vs. yes), and overweight/obesity status (body mass index [BMI] ≥ 25, no vs. yes). Healthcare-seeking behaviours were evaluated through routine physical exam participation (no vs. yes) and recent healthcare service utilization, including hospital admission in the previous year (no vs. yes) and outpatient care in the previous month (no vs. yes). When examining the correlates of agreement on hypertension control, we also included adherence to antihypertensive medication (no vs. yes) and regular BP monitoring (at least weekly at home or a healthcare centre, no vs. yes) [29], based on self-reported questionnaire survey data, as independent variables.

Household-level factors included household economic level, measured by household annual expenditure per capita and divided into quintiles ranging from the lowest (1st level) to the highest (5th level). Community-level variables comprised area of residence (urban vs. rural) and local economic level, determined by the Gross Domestic Product (GDP) per capita of the sampled city compared to the national average (underdeveloped vs. developed). Cities below the national average were classified as underdeveloped, while those above were categorized as developed.

### Statistical methods

The concordance between self-reported and clinically measured hypertensive conditions was evaluated using sensitivity, specificity, and Cohen’s kappa coefficient. Sensitivity, which assesses the proportion of TPs, indicates the ability of self-reported data to correctly identify individuals with biomedically confirmed hypertension diagnosis (or uncontrolled BP). High sensitivity suggests fewer FPs, demonstrating the effectiveness of self-reporting in identifying true cases. Specificity, on the other hand, measures the proportion of TNs and evaluates the capability of self-reported data to correctly exclude individuals without hypertension (or those with controlled high BP), with high specificity indicating fewer FNs. To assess the overall agreement between self-reported and objectively measured data on hypertensive conditions, Cohen’s kappa coefficient was employed. This statistical measure ranges from − 1 to 1, although it typically falls between 0 and 1, and quantifies the level of agreement between two raters across two categories or among unordered categorical variables with three or more categories [35, 36]. The kappa coefficient, which accounts for both observed agreement and agreement expected by chance [36], has been widely used in prior research to evaluate the validity or concordance between self-reported and clinically measured hypertension [17, 20, 37, 38]. In this study, the interpretation of kappa values followed the guidelines outlined by Landis and Koch [39]. These guidelines classify agreement levels as low (≤ 0.20), fair (0.21–0.40), moderate (0.41–0.60), substantial (0.61–0.80), and almost perfect or excellent (≥ 0.81). This classification was also applied to interpret sensitivity and specificity values. Each statistical measure, along with its 95% Confidence interval (CI), was calculated for the entire sample and further stratified by each independent variable included in the analysis.

To investigate the relationships between respondents’ characteristics and the agreement on hypertension diagnosis and control, both binary and multinomial logistic regression analyses were employed in this study. Binary logistic regression was performed to evaluate the likelihood of overall agreement, defined as either TP or TN outcomes, with adjusted odds ratios (AORs) calculated to determine the strength and direction of these associations. Multinomial logistic regression analysis was applied to identify factors associated with the relative-risk ratios (RRRs) of FP and FN self-reports separately, using overall agreement (TP or TN) as the reference category [40].

All statistical analyses were performed using Stata version 16.0 (Stata Corp., TX, USA). A significance level of *p* < 0.05 was set for all tests.

## Results

### Descriptive analysis

We identified 13,071 middle-aged and older adults for analysis. Descriptive statistics (see Table A1 in Additional File 2) indicate that 44.13% of the total samples were middle-aged, 45.56% were categorized as young-old, and 10.31% as old-old, with an average age of 61.50 years. Females represented 53.09% of the entire sample compared to 46.91% males. A substantial majority, 85.88%, were married. Educational levels varied, with 26.92% of the participants having no formal education, 41.50% only completing primary school, and 31.58% achieving secondary school education or higher. Employment status showed 36.58% of the respondents were not working or retired, while 38.77% worked in agriculture and 24.65% in non-agricultural jobs. Regarding the presence of multiple chronic diseases, 9.62% of the respondents reported having diabetes, while self-reported histories of CVD and CKD were 31.15% and 9.69%, respectively. In terms of behavioural risk factors, 27.63% were current smokers, 16.44% were former smokers, and 14.60% were classified as heavy drinkers. Most respondents had a BMI under 25, with 35.67% reporting being overweight or obese. Concerning healthcare-seeking behaviours, 41.89% participated in routine physical examinations, 14.43% had been admitted to the hospital in the past year, and 19.15% visited an outpatient doctor in the previous month. Among the 4734 respondents with self-reported hypertension, 2610 (55.13%) regularly took antihypertensive medications, while only 465 (3.56%) had their BP regularly monitored. Regarding household and community-level factors, our results indicated a range of household economic levels, with 18% falling into the lowest quintile and 15.22% into the highest. The majority of respondents (63.87%) lived in rural communities, whereas 36.13% resided in urban areas. Approximately one-third of the respondents came from developed cities (36.62%).

**Fig. 2:**
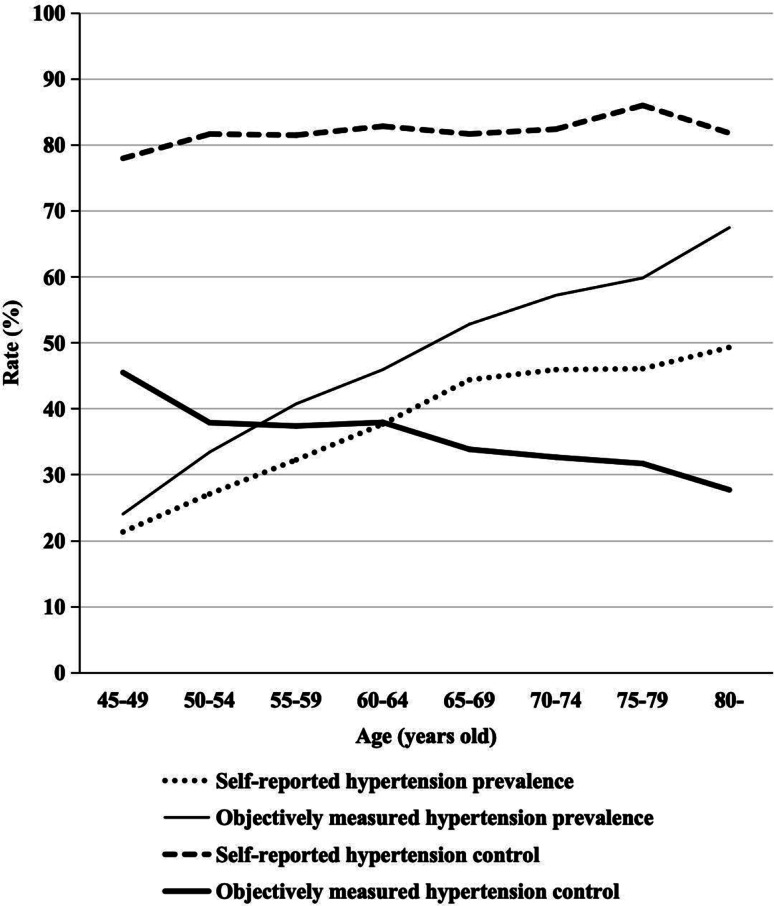
Discrepancy between self-reported and objectively measured hypertension diagnosis and control across age groups among middle-aged and older adults in China, 2015. Source: China Health and Retirement Longitudinal Study (CHARLS), 2015

Comparing the self-reported and objectively measured prevalences of hypertension, 4734 respondents (36.22%) of the total sample reported having a diagnosis of hypertension, but clinically measured BP values confirmed hypertension in 5838 individuals (44.66%). This discrepancy indicates that self-reports underestimated the actual prevalence of hypertension among middle-aged and older Chinese adults. The overall agreement rate on hypertension diagnosis between self-reported and clinical measurements was 80.93%. Figure 2 highlights a growing gap in hypertension prevalence between self-reports and clinical measurements with age.

In terms of hypertension control, among the 4734 respondents who reported having hypertension, 3884 (82.04%) claimed control of their BP condition through the self-reported questionnaire. However, only 1697 (35.85%) were confirmed to have controlled hypertension based on clinical measurements. This disparity points to a severe overestimation of hypertension control in self-reported data, with an overall agreement rate of only 44.63% with objective measurements. As depicted in Fig. 2, while self-reported control rates showed minimal variation with age, the actual control rates declined, indicating an increasing overestimation of hypertension control among older patients.

### Agreement between self-reported and objectively measured hypertension diagnosis

Table 1 presents the agreement parameters between self-reported and objectively measured hypertension diagnosis in the general sample. Compared to clinical measurements, self-reporting exhibited substantial sensitivity (69.20%, 95% CI: 66.87%, 71.51%), indicating that 30.80% of respondents were unaware of their hypertensive condition. The specificity was excellent at 90.41% (95% CI: 89.05%, 91.71%), indicating that only 9.59% of individuals mistakenly believed they had hypertension. The kappa coefficient was 0.60 (95% CI: 0.59, 0.62), representing moderate agreement between self-reported and objectively measured hypertension diagnosis. Subgroup analysis revealed kappa values ranging from 0.53 to 0.68, indicating moderate to substantial agreement in this regard, with higher values observed in urban residents, individuals with CKD, and those who took routine physical examinations or recently utilized healthcare services.

**Table 1:** Sensitivity, specificity, and kappa coefficient of self-reported hypertension diagnosis compared with objective measurements among middle-aged and older adults in China, 2015 (*n* = 13071)

Subgroup	Sensitivity % (95% CI)	Specificity % (95% CI)	Kappa coefficient (95% CI)
Overall	69.20(66.87, 71.51)	90.41 (89.05, 91.71)	0.60 (0.59, 0.62)
Age (years)			
Middle-aged (45–59)	65.91 (61.78, 69.97)	91.94 (90.20, 93.60)	0.61 (0.59, 0.62)
Young-old (60–74)	71.50 (68.32, 74.63)	88.69 (86.39, 90.88)	0.60 (0.58, 0.62)
Old-old (75 and older)	68.68 (62.48, 74.71)	88.82 (83.11, 93.95)	0.53 (0.49, 0.57)
Sex			
Female	71.73 (68.57, 74.84)	90.01 (88.12, 91.82)	0.63 (0.61, 0.64)
Male	66.44 (62.99, 69.85)	90.87 (88.91, 92.74)	0.58 (0.57, 0.60)
Marriage status			
Unmarried	68.60 (63.00, 74.06)	88.29 (83.76, 92.45)	0.55 (0.52, 0.58)
Married	69.33 (66.76, 71.87)	90.67 (89.25, 92.04)	0.61 (0.60, 0.63)
Educational level			
Illiteracy	69.52 (65.18, 73.77)	88.66 (85.73, 91.43)	0.59 (0.56, 0.61)
Primary school	70.43 (66.81, 73.97)	90.43 (88.32, 92.43)	0.62 (0.60, 0.64)
Secondary school and above	67.22 (62.88, 71.49)	91.71 (89.47, 93.82)	0.61 (0.59, 0.63)
Occupational status			
Without a job or retired	73.41 (70.09, 76.66)	89.74 (87.14, 92.21)	0.62 (0.60, 0.64)
Agricultural job	67.42 (63.36, 71.41)	90.07 (87.95, 92.10)	0.59 (0.57, 0.61)
Non-agricultural job	62.70 (57.25, 68.07)	91.59 (89.15, 93.87)	0.57 (0.55, 0.59)
Diabetes			
Without	67.23 (64.66, 69.78)	90.90 (89.54, 92.21)	0.60 (0.59, 0.61)
With	80.09 (74.84, 85.09)	81.04 (72.84, 88.60)	0.56 (0.53, 0.60)
CVD			
Without	61.47 (58.10, 64.80)	91.61 (90.20, 92.98)	0.56 (0.55, 0.57)
With	78.01 (74.93, 81.02)	85.10 (81.26, 88.73)	0.59 (0.57, 0.61)
CKD			
Without	68.16 (65.67, 70.63)	90.55 (89.14, 91.91)	0.60 (0.59, 0.61)
With	77.83 (71.30, 84.02)	88.91 (83.90, 93.45)	0.67 (0.63, 0.71)
Smoking			
Never	70.99 (67.88, 74.05)	89.95 (88.11, 91.71)	0.62 (0.61, 0.64)
Smoking quitter	71.45 (66.22, 76.54)	88.74 (84.79, 92.40)	0.60 (0.57, 0.63)
Current smoker	63.77 (59.00, 68.47)	92.12 (89.78, 94.31)	0.58 (0.56, 0.60)
Heavy drinking			
No	70.72 (68.22, 73.19)	90.59 (89.14, 91.99)	0.62 (0.61, 0.64)
Yes	60.91 (54.64, 67.09)	89.25 (85.35, 92.86)	0.51 (0.48, 0.54)
Overweight/obesity			
No	63.16 (59.83, 66.46)	91.74 (90.26, 93.17)	0.58 (0.56, 0.59)
Yes	76.17 (72.99, 79.28)	86.78 (83.77, 89.66)	0.61 (0.59, 0.63)
Routine physical examination			
No	64.26 (60.91, 67.58)	91.42 (89.79, 92.98)	0.58 (0.56, 0.59)
Yes	74.55 (71.36, 77.68)	88.67 (86.26, 90.97)	0.63 (0.61, 0.65)
Hospital admission			
No	67.21 (64.60, 69.79)	90.60 (89.17, 91.98)	0.59 (0.58, 0.61)
Yes	78.68 (73.63, 83.51)	89.00 (84.76, 92.90)	0.67 (0.64, 0.70)
Outpatient care			
No	66.98 (64.32, 69.61)	90.55 (89.06, 91.98)	0.59 (0.57, 0.60)
Yes	77.94 (73.22, 82.47)	89.77 (86.45, 92.86)	0.68 (0.65, 0.71)
Household economic level (quintile)			
1st (lowest)	65.49 (60.03, 70.85)	89.88 (86.76, 92.79)	0.57 (0.54, 0.59)
2nd	67.69 (63.39, 72.89)	90.82 (87.81, 93.62)	0.60 (0.57, 0.62)
3rd	68.95 (63.66, 74.11)	91.29 (88.32, 94.03)	0.61 (0.59, 0.64)
4th	71.70 (66.68, 76.58)	89.84 (86.64, 92.82)	0.62 (0.60, 0.65)
5th (highest)	71.97 (66.84, 76.95)	90.20 (87.06, 93.11)	0.63 (0.60, 0.66)
Area of residence			
Urban	70.47 (66.77, 74.11)	91.74 (89.54, 93.82)	0.63 (0.61, 0.65)
Rural	68.39 (65.38, 71.36)	89.72 (88.00, 91.38)	0.59 (0.58, 0.61)
Local economic level			
Underdeveloped	69.00 (66.03, 71.93)	90.41 (88.72, 92.04)	0.61 (0.59, 0.62)
Developed	69.53 (65.74, 73.25)	90.39 (88.10, 92.57)	0.61 (0.59, 0.63)

Source: China Health and Retirement Longitudinal Study (CHARLS), 2015

### Agreement between self-reported and objectively measured hypertension control

Table 2 presents the agreement parameters for self-reported versus clinically measured control of high BP among respondents who reported having hypertension. The sensitivity for self-reported hypertension control was notably low at 20.84% (95% CI: 18.05%, 23.71%), indicating that 79.16% of the hypertensive respondents incorrectly report their BP was under control. However, the specificity was excellent at 87.21% (95% CI: 84.01%, 90.24%), meaning only 12.79% of the respondents were unaware their high BP condition was controlled. The kappa coefficient was only 0.06 (95% CI: 0.05, 0.08), suggesting low agreement in this regard. Subgroup analysis showed kappa values ranging from 0.03 to 0.12, reinforcing the low agreement across subgroups. Notably, respondents who had been hospitalized in the previous year showed a slightly higher kappa value, exceeding 0.10, suggesting better concordance on the control of hypertensive condition among this group.

**Table 2:** Sensitivity, specificity, and kappa coefficient of self-reported hypertension control compared with objective measurements among middle-aged and older adults in China, 2015 (*n* = 4734)

Subgroup	Sensitivity % (95% CI)	Specificity % (95% CI)	Kappa coefficient (95% CI)
Overall	20.84 (18.05, 23.71)	87.21 (84.01, 90.24)	0.06 (0.05, 0.08)
Age (years)			
Middle-aged (45–59)	22.88 (17.84, 28.14)	86.90 (81.49, 91.86)	0.08 (0.05, 0.11)
Young-old (60–74)	20.52 (16.73, 24.45)	87.03 (82.51, 91.22)	0.06 (0.04, 0.08)
Old-old (75 and older)	17.53 (10.89, 24.72)	89.06 (79.60, 96.99)	0.04 (0.00, 0.09)
Sex			
Female	21.31 (17.47, 25.30)	86.46 (82.07, 90.56)	0.06 (0.04, 0.08)
Male	20.31 (16.29, 24.48)	88.20 (83.43, 92.57)	0.06 (0.04, 0.09)
Marriage status			
Unmarried	20.96 (14.47, 27.85)	86.21 (77.48, 93.89)	0.05 (0.01, 0.09)
Married	20.82 (17.74, 23.99)	87.40 (83.93, 90.66)	0.06 (0.05, 0.08)
Educational level			
Illiteracy	23.27 (18.01, 28.75)	85.84 (79.39, 91.71)	0.07 (0.04, 0.10)
Primary school	21.07 (16.70, 25.62)	85.71 (80.60, 90.47)	0.06 (0.03, 0.08)
Secondary school and above	18.05 (13.24, 23.13)	90.87 (85.49, 95.61)	0.07 (0.04, 0.10)
Occupational status			
Without a job or retired	19.93 (16.02, 24.00)	87.84 (82.88, 92.39)	0.06 (0.03, 0.08)
Agricultural job	22.51 (17.56, 27.67)	85.60 (80.09, 90.70)	0.07 (0.04, 0.10)
Non-agricultural job	20.25 (13.93, 26.98)	88.92 (82.06, 94.95)	0.08 (0.04, 0.11)
Diabetes			
Without	20.73 (17.64, 23.91)	87.83 (84.46, 91.00)	0.07 (0.05, 0.09)
With	21.34 (14.93, 28.13)	83.03 (72.71, 92.20)	0.03 (-0.01, 0.07)
CVD			
Without	20.15 (16.01, 24.47)	87.59 (83.60, 91.31)	0.07 (0.05, 0.10)
With	21.38 (17.64, 25.25)	86.55 (81.04, 91.60)	0.05 (0.03, 0.07)
CKD			
Without	20.72 (17.73, 23.79)	87.45 (84.10, 90.61)	0.06 (0.05, 0.08)
With	21.68 (13.98, 29.93)	85.03 (73.64, 94.84)	0.05 (0.00, 0.09)
Smoking			
Never	21.33 (17.58, 25.21)	86.47 (82.14, 90.52)	0.06 (0.04, 0.08)
Smoking quitter	19.48 (13.52, 25.81)	90.97 (84.17, 96.76)	0.08 (0.04, 0.11)
Current smoker	20.85 (15.23, 26.78)	86.14 (79.19, 92.40)	0.05 (0.02, 0.09)
Heavy drinking			
No	21.30 (18.25, 24.43)	87.31 (83.90, 90.53)	0.07 (0.05, 0.09)
Yes	18.22 (11.51, 25.47)	86.54 (76.77, 94.98)	0.03 (-0.01, 0.08)
Overweight/obesity			
No	23.00 (18.85, 27.29)	86.26 (81.81, 90.41)	0.08 (0.05, 0.10)
Yes	18.83 (15.12, 22.69)	88.42 (83.73, 92.71)	0.05 (0.03, 0.07)
Routine physical examination			
No	23.04 (18.90, 27.31)	86.37 (81.73, 90.69)	0.08 (0.05, 0.10)
Yes	18.77 (15.05, 22.64)	88.09 (83.59, 92.23)	0.05 (0.03, 0.07)
Hospital admission			
No	18.89 (15.90, 21.99)	87.29 (83.77, 90.61)	0.05 (0.03, 0.07)
Yes	28.81 (21.84, 36.05)	86.82 (78.79, 93.91)	0.12 (0.08, 0.16)
Outpatient care			
No	19.42 (16.34, 22.59)	87.71 (84.12, 91.07)	0.06 (0.04, 0.07)
Yes	25.81 (19.50, 32.39)	85.49 (78.18, 92.08)	0.09 (0.05, 0.13)
Antihypertensive medication adherence			
No	17.77 (12.78, 23.06)	88.68 (83.78, 93.13)	0.06 (0.03, 0.09)
Yes	21.97 (18.65, 25.39)	86.23 (81.95, 90.25)	0.06 (0.04, 0.08)
Regular BP monitoring			
No	20.36 (12.25, 29.14)	87.78 (75.67, 97.75)	0.05 (0.00, 0.10)
Yes	20.90 (17.94, 23.95)	87.16 (83.83, 90.32)	0.06 (0.05, 0.08)
Household economic level (quintile)			
1st (lowest)	19.50 (13.31, 26.11)	90.30 (83.55, 96.13)	0.08 (0.04, 0.12)
2nd	22.45 (16.00, 29.17)	84.10 (75.94, 91.46)	0.05 (0.01, 0.09)
3rd	22.59 (16.23, 29.30)	86.83 (79.12, 93.68)	0.07 (0.03, 0.11)
4th	21.10 (15.15, 27.39)	84.89 (77.32, 91.73)	0.05 (0.01, 0.08)
5th (highest)	18.55 (12.75, 24.73)	89.92 (83.34, 95.64)	0.07 (0.03, 0.10)
Area of residence			
Urban	17.14 (13.08, 21.40)	90.47 (85.58, 94.84)	0.05 (0.03, 0.08)
Rural	23.29 (19.55, 27.14)	85.44 (81.23, 89.40)	0.07 (0.05, 0.09)
Local economic level			
Underdeveloped	23.49 (19.80, 27.29)	85.34 (81.03, 89.39)	0.07 (0.05, 0.09)
Developed	16.48 (12.38, 20.80)	90.31 (85.57, 94.57)	0.05 (0.03, 0.08)

Source: China Health and Retirement Longitudinal Study (CHARLS), 2015

### Factors associated with agreement between self-reported and objectively measured hypertension diagnosis

Table 3 displays the estimated effects of respondents’ characteristics on the agreement between self-reported and objectively measured hypertension diagnosis in the general sample. Column 1 presents the AORs and their 95% CIs for the associations with overall agreement, calculated using binary logistic regression. Columns 2 and 3 provide the RRRs and their 95% CIs for the associations with FP and FN reporting on hypertensive diagnosis, respectively, based on multinomial logistic regression models.

The results showed that, compared to middle-aged individuals, young-old (AOR = 0.82, 95% CI: 0.74, 0.91) and old-old respondents (AOR = 0.68, 95% CI: 0.57, 0.81) had lower odds of the overall agreement. This reduced likelihood of agreement among the young-old and old-old groups was primarily due to a 30% and 72% increase, respectively, in the risk of FN reporting (*p* < 0.001). Males were less likely to accurately report hypertension compared to females; however, this difference was not statistically significant (*p* > 0.05). In contrast, males had a significantly 19% higher rate of FN reporting on hypertension diagnosis compared to females (*p* < 0.05). Additionally, married respondents were more likely to be in agreement on hypertension diagnosis than unmarried ones (AOR = 1.23, 95% CI: 1.08, 1.40). The multinomial logistic regression results showed a 23% decrease in the risk of FN reporting among married respondents (*p* < 0.001), despite no significant difference in the risks of FP reporting between married and unmarried individuals (*p* > 0.05). Concerning educational attainment, respondents with a primary education were more likely to align with clinically measured hypertension diagnosis than those who were illiterate (AOR = 1.14, 95% CI: 1.02, 1.28). The increased probability of overall agreement was mainly attributed to a significant decrease in the risk of FN reporting (RRR = 0.87, 95% CI: 0.76, 0.99). However, having an educational level of secondary school or above did not show a significant association with the overall agreement (*p* > 0.05). Furthermore, no significant relationships were observed between the agreement on hypertension diagnosis and occupational status (*p* > 0.05), as indicated by both binary and multinomial logistic regression analyses.

Regarding multimorbidity, respondents with a self-reported history of CKD had higher odds of the overall agreement on hypertension diagnosis compared to those without (AOR = 1.19, 95% CI: 1.02, 1.39). This increased likelihood was mainly due to a 24% decrease in the risk of FN reporting (*p* < 0.01). While the presence of diabetes or CVD did not show a statistically insignificant association with the agreement (*p* > 0.05), the risk of FN reporting was significantly higher in the presence of CVD (RRR = 1.21, 95% CI: 1.08, 1.36). Behavioural risk factors such as smoking and overweight/obesity were not significantly related to the overall agreement (*p* > 0.05). However, heavy drinking was associated with lower odds of the agreement (AOR = 0.71, 95% CI: 0.62, 0.80). The multinomial logistic regression results further indicated that the lower likelihood of agreement among heavy drinkers was primarily due to a significantly higher rate of FN reporting (RRR = 1.47, 95% CI: 1.28, 1.70) compared to non-drinkers. Concerning healthcare-seeking behaviour, binary logistic regression results showed that recent use of inpatient (AOR = 1.20, 95% CI: 1.05, 1.38) or outpatient care (AOR = 1.28, 95% CI: 1.14, 1.45) was linked to significantly higher odds of the overall concordance between self-reported and clinically measured hypertensive diagnosis. This increased likelihood was primarily reflected in a significant 21% (*p* < 0.01) and 29% (*p* < 0.001) decrease, respectively, in the risk of FN reporting, as indicated by multinomial logistic analysis. Additionally, participants in routine physical examinations had a significant decrease in the risk of FN reporting (RRR = 0.86, 95% CI: 0.77, 0.95) compared to non-participants, although routine physical examinations showed no significant relationship with the overall agreement (*p* > 0.05).

As for household and community-level factors, the household economic level was positively associated with the concordance between self-reported and objectively measured hypertension diagnosis. Respondents in the middle-high and highest economic quintiles showed not only greater odds of overall agreement (AOR for middle-high = 1.14, 95% CI: 1.00, 1.31; AOR for highest = 1.18, 95% CI: 1.02, 1.36) but also a lower risk of FN reporting (RRR for the middle-high = 0.84, 95% CI: 0.71, 0.98; RRR for the highest = 0.80, 95% CI: 0.68, 0.95) compared to those in the lowest quintile. Additionally, no statistically significant associations were observed between the overall agreement and residential area or local economic level (*p* > 0.05). Compared to those living in urban areas, rural residents had a higher risk of FP reporting on hypertension diagnosis, with an estimated RRR of 1.35 (95% CI: 1.13, 1.63).

**Table 3:** Factors associated with agreement between self-reported and objectively measured diagnosis of hypertension among middle-aged and older adults in China, 2015 (*n* = 13071)

Variable	Agreement ^b^	FP VS Agreement ^c^	FN VS Agreement ^c^
**Individual-level factor**			
*Demographic and socioeconomic factor*			
Age (years)			
Middle-aged (45–59) ^a^	1.00	1.00	1.00
Young-old (60–74)	0.82^***^ (0.74,0.91)	1.09 (0.91, 1.30)	1.30^***^ (1.15, 1.46)
Old-old (75 and older)	0.68^***^ (0.57,0.81)	0.89 (0.63, 1.24)	1.72^***^ (1.42, 2.09)
Sex			
Female ^a^	1.00	1.00	1.00
Male	0.89 (0.77,1.02)	0.99 (0.78, 1.28)	1.19^*^ (1.01, 1.39)
Marriage			
Unmarried ^a^	1.00	1.00	1.00
Married	1.23^**^ (1.08,1.40)	0.93 (0.73, 1.18)	0.77^***^ (0.67, 0.89)
Educational level			
Illiteracy ^a^	1.00	1.00	1.00
Primary school	1.14^*^ (1.02,1.28)	0.91 (0.74, 1.10)	0.87^*^ (0.76, 0.99)
Secondary school and above	1.05 (0.92,1.20)	0.86 (0.68, 1.10)	0.99 (0.85, 1.16)
Occupation			
Without a job or retired ^a^	1.00	1.00	1.00
Agricultural job	0.98 (0.87,1.10)	1.21 (0.99, 1.48)	0.95 (0.83, 1.08)
Non-agricultural job	0.93 (0.82,1.06)	1.20 (0.95, 1.51)	1.02 (0.88, 1.18)
*Multimorbidity*			
Diabetes			
Without ^a^	1.00	1.00	1.00
With	0.92 (0.79,1.08)	1.10 (0.84, 1.44)	1.07 (0.90, 1.28)
CVD			
Without ^a^	1.00	1.00	1.00
With	0.90 (0.82,1.01)	0.92 (0.77, 1.10)	1.21^**^ (1.08, 1.36)
CKD			
Without ^a^	1.00	1.00	1.00
With	1.19^*^ (1.02,1.39)	1.05 (0.81, 1.36)	0.76^**^ (0.63, 0.92)
*Behavioural risk*			
Smoking			
Never ^a^	1.00	1.00	1.00
Smoking quitter	1.02 (0.88,1.19)	0.96 (0.73, 1.26)	0.98 (0.82, 1.17)
Current smoker	1.08 (0.94,1.24)	0.78 (0.60, 1.01)	0.99 (0.84, 1.16)
Heavy drinking			
No^a^	1.00	1.00	1.00
Yes	0.71^***^ (0.62,0.80)	1.25 (0.99, 1.58)	1.47^***^ (1.28, 1.70)
Overweight/obesity			
No ^a^	1.00	1.00	1.00
Yes	0.92 (0.83,1.01)	1.10 (0.93, 1.30)	1.09 (0.97, 1.21)
*Healthcare-seeking behaviour*			
Routine physical examination			
No ^a^	1.00	1.00	1.00
Yes	1.08 (0.98,1.19)	1.11 (0.94, 1.31)	0.86^**^ (0.77, 0.95)
Hospital admission			
No ^a^	1.00	1.00	1.00
Yes	1.20^**^ (1.05,1.38)	0.93 (0.74, 1.17)	0.79^**^ (0.67, 0.92)
Outpatient care			
No ^a^	1.00	1.00	1.00
Yes	1.28^***^ (1.14,1.45)	0.95 (0.78, 1.16)	0.71^***^ (0.62, 0.82)
**Household-level factor**			
Household economic level (quintile)			
1st (lowest) ^a^	1.00	1.00	1.00
2nd	1.07 (0.94, 1.24)	0.89 (0.70, 1.13)	0.94 (0.81, 1.10)
3rd	1.13 (0.99, 1.30)	0.85 (0.66, 1.09)	0.89 (0.76, 1.04)
4th	1.14^*^ (1.00, 1.31)	0.98 (0.77, 1.25)	0.84^*^ (0.71, 0.98)
5th (highest)	1.18^*^ (1.02, 1.36)	0.99 (0.78, 1.27)	0.80^**^ (0.68, 0.95)
**Community-level factor**			
Area of residence			
Urban ^a^	1.00	1.00	1.00
Rural	0.94 (0.85, 1.04)	1.35^**^ (1.13, 1.63)	0.98 (0.87, 1.10)
Local economic level			
Underdeveloped ^a^	1.00	1.00	1.00
Developed	0.95 (0.86, 1.04)	1.02 (0.86, 1.20)	1.06 (0.95, 1.18)
Wald/LR chi2	147.60^***^	246.27^***^	
Pseudo R2	0.0114	0.0157	
Number of observations	13,071	13,071	

Source: China Health and Retirement Longitudinal Study (CHARLS), 2015

Note. ^a^ Reference group. ^b^ Results are presented as AOR with 95% CI in parentheses. ^c^ Results are presented as RRR with 95% CI in parentheses. ^*^p < 0.05, ^**^p < 0.01, ^***^p < 0.001

### Factors associated with agreement between self-reported and objectively measured hypertension control

Table 4 presents factors relating to the agreement between self-reported and objectively measured control of hypertension among respondents who reported having hypertension, based on binary logistic regression (column 1) and multinomial logistic regression analyses (columns 2 and 3). The results showed that old-old patients were significantly less likely to have agreement compared to their middle-aged counterparts (AOR = 0.72, 95% CI: 0.57, 0.90). This decrease in the likelihood was primarily attributed to a significant higher risk of FN reporting (RRR = 1.48, 95% CI: 1.17, 1.86). However, no significant associations were found between the agreement with other demographic and socioeconomic variables, including sex, marital status, education, and occupational status (*p* > 0.05).

Self-reported combinations of diabetes (AOR = 0.83, 95% CI: 0.70, 0.98) and CVD (AOR = 0.65, 95% CI: 0.57, 0.73) were negatively associated with the likelihood of the overall concordance between self-reported and clinically measured BP control, suggesting that multimorbidity may complicate accurate self-reporting of hypertension control. Specifically, patients with diabetes had an 18% increase in the risk of FN reporting (*p* < 0.05), and those with CVD had a 64% increase in the risk of FN reporting (*p* < 0.001), compared to those without multimorbidity. However, no significant relationship was observed between CKD and the agreement on hypertension control (*p* > 0.05). Additionally, certain behavioural risks were linked to the overall agreement. Heavy drinking (AOR = 0.72, 95% CI: 0.60, 0.87) and having a BMI over 25 (AOR = 0.79, 95% CI: 0.70, 0.89) were associated with significantly lower odds of consistent self-reported and measured hypertension control statuses. The reduced likelihood of the agreement for heavy drinker and patients who were overweight or obese was primarily due to a 41% and 32% increase, respectively, in the risk of FN reporting (*p* < 0.001). However, smoking status showed no significant association with the agreement on hypertension control (*p* > 0.05).

Recent healthcare utilization showed a positive association with the agreement. Specifically, patients who had outpatient visits in the past month (AOR = 1.18, 95% CI: 1.02, 1.36) or were hospitalized in the past year (AOR = 1.36, 95% CI: 1.17, 1.59) were more likely to have concordant reports on hypertension control. The risk of FN reporting for those who received inpatient and outpatient care was 28% (*p* < 0.001) and 17% (*p* < 0.05) lower, respectively, compared to those who did not utilize these services. Contrarily, regular users of antihypertensive medications were less likely to achieve the agreement (AOR = 0.80, 95% CI: 0.70, 0.91), with a 28% increase in the risk of FN reporting (*p* < 0.001). Moreover, there was no significant association between routine physical examination or regular BP monitoring and the agreement, as well as with the risk of FP or FN reporting (*p* > 0.05).

Regarding household and community-level factors, no statistically significant associations were observed between the agreement on high BP control and household economic level, area of residence, or local economic level (*p* > 0.05). However, the multinomial logistic regression results indicated that, compared to those in the lowest quintile of household economic level, patients in the middle-low and middle-high economic quintiles had a 72% and 77% higher risks of FP reporting on hypertension control, respectively (*p* < 0.05).

**Table 4:** Factors associated with agreement between self-reported and objectively measured control of hypertension among middle-aged and older adults in China, 2015 (*n* = 4734)

Variable	Agreement	FP VS Agreement ^c^	FN VS Agreement ^c^
**Individual-level factor**			
*Demographic and socioeconomic factor*			
Age (years)			
Middle-aged (45–59) ^a^	1.00	1.00	1.00
Young-old (60–74)	0.89 (0.77,1.03)	0.89 (0.64, 1.25)	1.15 (0.99, 1.33)
Old-old (75 and older)	0.72^**^ (0.57,0.90)	0.72 (0.40, 1.28)	1.48^**^ (1.17, 1.86)
Sex			
Female ^a^	1.00	1.00	1.00
Male	0.89 (0.74,1.07)	0.89 (0.56, 1.39)	1.14 (0.95, 1.38)
Marriage			
Unmarried ^a^	1.00	1.00	1.00
Married	1.10 (0.93,1.31)	0.98 (0.66, 1.48)	0.90 (0.75, 1.07)
Educational level			
Illiteracy ^a^	1.00	1.00	1.00
Primary school	1.08 (0.93,1.25)	1.21 (0.85, 1.71)	0.90 (0.77, 1.05)
Secondary school and above	1.02 (0.85,1.22)	0.78 (0.49, 1.24)	1.00 (0.83, 1.20)
Occupation			
Without a job or retired ^a^	1.00	1.00	1.00
Agricultural job	1.04 (0.90,1.20)	1.01 (0.72, 1.42)	0.96 (0.83, 1.11)
Non-agricultural job	1.09 (0.92,1.30)	0.95 (0.61, 1.47)	0.91 (0.76, 1.09)
*Multimorbidity*			
Diabetes			
Without ^a^	1.00	1.00	1.00
With	0.83^*^ (0.70,0.98)	1.40 (0.95, 2.07)	1.18^*^ (1.00, 1.40)
CVD			
Without ^a^	1.00	1.00	1.00
With	0.65^***^ (0.57,0.73)	0.84 (0.62, 1.14)	1.64^***^ (1.44, 1.86)
CKD			
Without ^a^	1.00	1.00	1.00
With	0.87 (0.72,1.05)	1.12 (0.71, 1.75)	1.15 (0.95, 1.39)
*Behavioural risk*			
Smoking			
Never ^a^	1.00	1.00	1.00
Smoking quitter	1.08 (0.89,1.31)	0.70 (0.42, 1.19)	0.94 0(0.77, 1.15)
Current smoker	1.04 (0.86,1.26)	1.04 (0.65, 1.67)	0.96 (0.78, 1.17)
Heavy drinking			
No ^a^	1.00	1.00	1.00
Yes	0.72^**^ (0.60,0.87)	1.09 (0.69, 1.72)	1.41^***^ (1.16, 1.71)
Overweight/obesity			
No ^a^	1.00	1.00	1.00
Yes	0.79^***^ (0.70,0.89)	0.82 (0.61, 1.10)	1.32^***^ (1.17, 1.50)
*Healthcare-seeking behaviour*			
Routine physical examination			
No ^a^	1.00	1.00	1.00
Yes	0.95 (0.84,1.07)	1.00 (0.75, 1.34)	1.06 (0.93, 1.19)
Hospital admission			
No ^a^	1.00	1.00	1.00
Yes	1.36^**^ (1.17,1.59)	0.84 (0.57, 1.23)	0.72^***^ (0.62, 0.85)
Outpatient care			
No ^a^	1.00	1.00	1.00
Yes	1.18^*^ (1.02,1.36)	1.06 (0.76, 1.48)	0.83^*^ (0.72, 0.96)
Antihypertensive medication adherence			
No ^a^	1.00	1.00	1.00
Yes	0.80^**^ (0.70, 0.91)	1.06 (0.78, 1.44)	1.28^***^ (1.12, 1.47)
Regular BP monitoring			
No ^a^	1.00	1.00	1.00
Yes	0.90 (0.74, 1.11)	0.96 (0.55, 1.66)	1.11 (0.90, 1.37)
**Household-level factor**			
Household economic level (quintile)			
1st (lowest) ^a^	1.00	1.00	1.00
2nd	0.96 (0.79, 1.16)	1.72^*^ (1.08, 2.73)	0.99 (0.82, 1.20)
3rd	0.99 (0.82, 1.20)	1.40 (0.86, 2.27)	0.98 (0.80, 1.18)
4th	0.97 (0.80, 1.17)	1.77^*^ (1.11, 2.82)	0.98 (0.81, 1.18)
5th (highest)	1.09 (0.90, 1.33)	1.27 (0.76, 2.13)	0.88 (0.72, 1.08)
**Community-level factor**			
Area of residence			
Urban ^a^	1.00	1.00	1.00
Rural	1.10 (0.97, 1.26)	1.37 (0.97, 1.93)	0.87 (0.76, 1.00)
Local economic level			
Underdeveloped ^a^	1.00	1.00	1.00
Developed	0.93 (0.82, 1.06)	0.74 (0.53, 1.02)	1.11 (0.97, 1.26)
Wald/LR chi2	157.14^***^	263.21^***^	
Pseudo R2	0.0255	0.0329	
Number of observations	4734	4734	

Source: China Health and Retirement Longitudinal Study (CHARLS), 2015

Note. ^a^ Reference group. ^b^ Results are presented as AOR with 95% CI in parentheses. ^c^ Results are presented as RRR with 95% CI in parentheses. ^*^p < 0.05, ^**^p < 0.01, ^***^p < 0.001

## Discussion

Accurate and reliable estimates of hypertension prevalence and management are essential for monitoring CVD risks and informing community-based public health decision-making, particularly in aging societies [8]. This study provides critical insights into the agreement and its associated factors between self-reported and objectively measured data, not only on the diagnosis but also on the control of hypertension among middle-aged and older adults in China, offering a nationwide representative perspective. Our findings revealed moderate agreement on hypertension diagnosis but only low concordance on hypertension control when comparing self-reported data with biometric measurements. This discrepancy, on the one hand, highlights a significant underestimation of hypertension prevalence through self-reporting among middle-aged and older Chinese adults, consistent with prior studies indicating an awareness gap between self-reports and actual prevalence [20, 41]. Despite the excellent specificity in self-reported hypertension diagnosis, which mirrors similar situations in both developed and developing countries [37, 38, 42–44], the issue of unawareness or under-diagnosis of hypertension among the older population persists as a critical public health concern. In our study, approximately 30% of middle-aged and older adults were unaware of their hypertensive condition, primarily due to the limited sensitivity of self-reporting. Previous studies have indicated that poor sensitivity of self-reporting often results from inadequate BP screening, which may be linked to insufficient access to or poor quality of healthcare services [17, 22, 43]. Consequently, asymptomatic hypertension in its early phase may remain undetected. This finding demonstrates the urgent need for governments to strengthen public health strategies for improving hypertension detection and screening. It also emphasizes the importance of encouraging older adults to adopt self-management practices for NCDs, such as regularly monitoring of BP and blood glucose levels at home, actively participating in health check-ups or recommended screenings, and fostering healthy behaviours and lifestyles.

On the other hand, the present study demonstrated a significant overestimation of hypertension control through self-reporting. Our results showed a sensitivity of 20.84% (95%CI: 18.05%, 23.71%), indicating that only one in five middle-aged and older hypertensive patients with uncontrolled hypertension in China accurately self-reported their condition, while the remaining 80% mistakenly believed their BP was under control. This low sensitivity, substantially lower than the approximately 80% reported in developed countries such as Canada [29], suggests an important issue with FN self-reporting, where the majority of patients with uncontrolled hypertension are unaware of their condition. A possible contributing factor to this discrepancy could be patients’ limited health literacy, which is often associated with lower educational attainment [45]. This was evident in our sample, where over a quarter of among middle-aged and older respondents were illiterate, and the majority had only completed primary education. This lack of health understanding may lead to misconceptions about hypertension control, such as confusing “*controlled*” disease with “*being without apparent symptoms*”, relying on subjective feeling rather than self-monitoring to report chronic conditions, or unknowing of normal BP levels [16]. As a result, hypertension, often asymptomatic, might be perceived as controlled until severe symptoms like headaches or dizziness occur.

Identifying factors associated with agreement on hypertension is crucial for its accurate diagnosis and effective management [41]. Regarding individual-level demographic factors, we initially observed an increasing disparity between self-reported and clinically measured hypertension with advancing age. Older adults, particularly the old-old, were significantly less likely to report accurate diagnoses or control of hypertension compared to middle-aged individuals. The discrepancy between self-reported data and biomedical measurements was mainly attributed to older adults being more prone to FN reports for both hypertension diagnosis and control, suggesting a tendency for unawareness of their hypertensive status. This finding is in line with previous studies indicating that the reliability of self-reported data decreases with age across various chronic conditions [21, 38, 46, 47]. This trend highlights the importance of considering potential memory or recall biases when evaluating self-reported responses from older people [41, 48]. They may not reliably interpret their health symptoms, such as headaches, as indicators of high BP but rather as normal aspects of aging. Additionally, we found that married individuals were more likely to accurately report hypertension diagnoses than their unmarried peers, as evidenced by a significant reduction in the risk of FN reports. This outcome is likely due to better health monitoring and support from their spouses, a trend similarly observed in other developing countries [47], where marital relationships contribute to a greater awareness of one’s health status.

Investigation into the role of socioeconomic factors in the observed discrepancies revealed that, compared to those who were illiterate or from poorer households, respondents with formal education or higher family economic levels were more likely to accurately report their hypertension diagnosis, showing a significantly reduced risk in FN reporting. This aligns with previous findings indicating that individuals with higher levels of education or greater economic status are typically more cognizant of chronic conditions such as hypertension or diabetes, largely due to their enhanced health literacy, more consistent monitoring practices, and earlier access to diagnoses [22]. However, this association of socioeconomic status was not apparent with the agreement on hypertension control, indicating the need for further exploration of other potential factors. Meanwhile, although no significant disparity in the likelihood of the overall agreement on hypertension diagnosis was found between rural and urban respondents, rural residents exhibited a higher risk of FP reporting, suggesting poor validity of self-reported hypertension among this group. This finding is in line with earlier studies, which indicate that rural residents often demonstrate less accuracy in self-reporting due to limited access to continuous monitoring and professional diagnosis, and insufficient knowledge of hypertension symptoms and diagnostic criteria compared to their rural counterparts [17, 20, 40]. Despite improvements in rural primary healthcare services since China’s New Healthcare Reform in 2009 [49], the discrepancy in healthcare resource accessibility and chronic disease detection between urban and rural areas remains a significant concern.

Given the high prevalence of multimorbidity among middle-aged and older adults in China [50], this study also investigated the relationship between the presence of multiple chronic diseases and the agreement between self-reporting and biomarkers on hypertensive conditions. Our results suggest that respondents with CKD were more likely to achieve agreement on hypertension diagnosis, largely attributed to a lower risk of FN reporting compared to those without the condition. This is possibly due to the increased exposure of comorbid patients to monitoring programs, more frequent interactions with healthcare professionals, and better access to health information [19]. Conversely, having a combination of diabetes or CVD was associated with a lower likelihood of agreement on hypertension control. Specifically, hypertensive patients combined with CVD or diabetes were at a significantly higher risk of FN reporting compared to those without multimorbidity, as they were more likely to incorrectly perceive their BP as being controlled. Two factors may contribute to this discrepancy. First, symptoms of CVD, such as chest pain and dizziness, often overlap with those of hypertension, leading patients to attribute these symptoms primarily to their heart condition, thus overestimating their hypertension control. Second, managing multiple chronic conditions adds complexity to patients’ ability to accurately access and report their health status. In particular, patients with diabetes and cardiovascular conditions are more prone to BP fluctuations, which may make self-reporting of hypertension control less accurate [51]. These findings highlight the necessity for regular follow-up appointments specifically designed for middle-aged and older patients with multiple chronic diseases.

In examining the associations of behavioural risk factors and the accuracy of hypertension self-reporting, we found that heavy drinking was associated with a significantly lower likelihood of agreement in both hypertension diagnosis and control. Nearly 40% of middle-aged and older hypertensive patients who were heavy drinkers were unaware of their condition, indicating a higher risk of FN reporting in hypertension diagnosis. Furthermore, over 91% of hypertensive patients who drank heavily incorrectly reported their BP as being under control, further highlighting an elevated risk of FN reporting in hypertension management among this group. This may be partly due to the adverse effects of excessive alcohol consumption on the cardiovascular system, which leads to fluctuations in BP and complicates accurate self-reporting of hypertensive conditions [52]. Moreover, since alcohol initially lowers BP for up to 12 h after ingestion, followed by a subsequent increase [53], many individuals mistakenly believe that alcohol consumption helps managing high BP, while disregarding the long-term risks of excessive drinking. Based on our routine observations, this misconception seems particularly prevalent among middle-aged and older Chinese males. They may hold cognitive biases about the impact of alcohol on BP, thus compromising the reliability of their self-reported hypertensive condition. Meanwhile, previous research has indicated that heavy drinking is often associated with other unhealthy lifestyle choices, such as irregular dietary habits and lack of physical activity [20, 22], further disrupting BP regulation and reducing the accuracy of self-reported hypertension. This is supported by our findings that being overweight or obese was associated with a lower likelihood of agreement on hypertension control, consistent with previous research linking higher BMI with decreased health awareness and worse chronic conditions [38, 43, 47].

In terms of healthcare-seeking behaviours, we found that respondents who had recently utilized outpatient or inpatient services were more likely to accurately report both their hypertension diagnosis and control status. Specifically, recent engagement of healthcare services was related to a reduced risk of FN reporting for both hypertension diagnosis and control, suggesting that regular healthcare contact enhances patients’ ability to recognize and accurately report uncontrolled high BP. This finding is consistent with previous research, likely due to enhanced detection and monitoring of health conditions resulting from their frequent interactions with healthcare providers, which in turn improves the validity of self-reported hypertension [19, 22, 43]. This phenomenon may also explain the differences in the sensitivity of self-reported hypertension diagnosis between males and females in our study. Previous studies have shown that men are generally less likely to seek medical advice or visit healthcare facilities compared to women, who tend to have more frequent healthcare interactions due to reproductive health needs [54]. Meanwhile, under-diagnosis of chronic diseases often stems from barriers in accessing healthcare services [17]. As a result, women may have better awareness of their health status and are therefore more inclined to provide accurate self-reports of their chronic conditions.

The present study also found a significant reduction in the risk of FN reporting for hypertension diagnosis among middle-aged and older Chinese adults who received preventive care services, particularly routine physical examinations. This finding is in line with prior studies and highlights the benefits of regular health checkups in improving hypertension awareness [24, 55]. However, no association was observed between the overall agreement on hypertension control and the use of routine physical examination or regular BP monitoring, suggesting that these preventive care services may have a limited impact on hypertension management. This may be attributed to the limited quality and uneven distribution of such services in China. Although individuals may undergo regular health check-ups, they may not receive sufficient follow-up care or medical guidance for managing chronic diseases [56], which could reduce the effectiveness of preventive service utilization in enhancing the accuracy of self-reports.

Lastly, our findings indicated a reduced likelihood of concordance between self-reported and objectively measured BP control among middle-aged and older patients who reported adhering to antihypertensive medication, compared to those who did not take the medication regularly. This discrepancy was primarily driven by an increased risk of FN reporting on hypertension control. Importantly, these findings do not diminish the well-established importance of medication adherence in hypertension management, but rather suggest that patients adhering to treatment may exhibit over-confidence in the efficacy of their medication. In China, the BP control rate among hypertensive adults on medication is less than 40% [57]. Despite the availability and widespread use of low-cost generic antihypertensive medications with generally good adherence [58], the high prevalence of uncontrolled hypertension among patients who report adherence remains a significant public health challenge [59]. This discrepancy between self-reports and clinical measurements appears to be multifactorial. First, low health literacy and limited health awareness among middle-aged and older Chinese adults, particularly those with chronic conditions, contribute to inadequate self-management of hypertension [45]. Many patients lack a comprehensive understanding of proper medication use and the broader importance of lifestyle modifications [60]. Some may overestimate the effectiveness of medication, mistakenly believing that simply adhering to their prescription is sufficient for BP control, while neglecting other essential factors such as diet, physical activity, and mood management. This can contribute to biased self-assessments and inaccurate reporting of hypertension control. Additionally, the insufficiency of consistent and high-quality disease monitoring and inadequate medication guidance from community healthcare services in China further hinder effective hypertension management and the accuracy of self-reported data. Limited access to timely follow-ups, personalized treatment adjustments, and patient education exacerbates this issue [58, 59], leaving many patients without the necessary support to manage their condition effectively. As a result, the gap between perceived and actual BP control widens, contributing to both poor health outcomes and unreliable self-reporting. These findings highlight the urgent need to enhance the quality of primary public health service and promote the broader use of person-centred combination therapy [61] to ensure valid self-reporting and foster effective self-management of hypertensive and other chronic conditions.

### Implications

This study offers several implications for public health researchers and policymakers. Firstly, given the observed discrepancies between self-reported and clinically measured hypertensive conditions, integrating questionnaire surveys with clinical measurements in public health research could help improve the accuracy of disease burden estimates and health technology assessments. Secondly, there is a need for the Chinese government to enhance the accessibility and quality of community-based public health services for older population. Targeting resources towards specific groups, such as the socioeconomically disadvantaged and the old-old adults, may encourage broader participation in regular health screenings and disease monitoring. Furthermore, comprehensive disease management programs focusing on patients with multiple chronic conditions are needed, including regular follow-up appointments with family doctors, encouragement of self-monitoring, and provision of person-centred medical services and medication guidance. Lastly, given the importance of cultivating healthy lifestyle and effective medication adherence in managing chronic conditions, initiatives to promote health behaviours and improve medication literacy through health promotion and medical education should be strengthened. Collectively, these strategies can inform public health policy and enhance community public health practices, potentially improving the management of hypertension and other chronic conditions in aging populations.

### Strengths and limitations

This study offers an important examination on the agreement between self-reported and objectively measured hypertension, addressing both diagnosis and control, among middle-aged and older Chinese adults. To the best of our knowledge, this is the first investigation to integrate large-scale, community-based questionnaire survey data with health examination data to evaluate the accuracy of self-reported hypertension control in this demographic. By extending previous studies that primarily focused on the validity of self-reported hypertension diagnosis, we offer a broader evaluation of both diagnosis and control using a nationally representative sample. Incorporating individual, household and community-level variations strengthens the relevance of our findings to the middle-aged and older population in China. Additionally, BP values in our study were measured three times for each respondent to reduce the risk of FP reporting, aligning with clinical best practices for accuracy [38].

Despite the strengths of this study, several limitations should be acknowledged. Firstly, potential misclassifications due to white-coat syndrome or undetected nocturnal hypertension [19] may have introduced bias into our estimates. Secondly, since participation in the physical examinations conducted by the CHARLS team was voluntary, the exclusion of respondents with missing biomarker data might have led to sample selection bias. Of the sample in the CHARLS dataset, approximately 23% of the interviewees had missing anthropometric measurements. While the overall response rate was relatively high (exceeding 80%), the valid response rate for BP measurements was 63%, which could potentially have caused selection bias. Specifically, women were more likely to participate in physical examinations, whereas younger men were underrepresented, possibly due to their work commitments [20, 31]. Additionally, individuals in very poor health status were also less likely to complete the physical examinations [31], potentially introducing estimation bias. However, given that CHARLS respondents are fairly evenly distributed across various demographic and socioeconomic background characteristics and that strict quality control measures were applied throughout the study [20], our findings retain a certain level of generalizability to the middle-aged and older population in China. Future studies may consider matching survey data with official health monitoring data from health departments or healthcare facilities to create a quota sample, thereby addressing potential voluntary selection biases. Thirdly, although we have controlled for factors at the individual, household, and community levels, other relevant factors, such as family history of hypertension and dietary habits, were not included due to data limitations. Future research could incorporate these factors when available to build more robust models of agreement between self-reported and biomedically measured hypertension. Lastly, the cross-sectional design of this study limits our ability to draw causal inferences about the observed relationships. Longitudinal studies with follow-up data would be beneficial in addressing this limitation and allow for more comprehensive analyses of BP trends over time.

## Conclusions

This study assessed the agreement between self-reported and objectively measured hypertensive conditions among middle-aged and older Chinese adults. Self-reports tended to underestimate hypertension prevalence and overestimated control rates, highlighting the challenges of relying solely on self-reported data for public health assessments. This study further established the associations between agreement on hypertension and various individual, household and community-level factors. Older age and heavy drinking were associated with a lower likelihood of agreement on hypertension diagnosis, while factors such as being married, having primary education, presence of CKD, recent outpatient or inpatient service utilization, and higher household economic status were linked to a higher likelihood of the agreement. For patients who reported having a hypertension diagnosis, older age, the presence of comorbid diabetes or CVD, heavy drinking, being overweight or obese, and medication adherence were identified as factors associated with a lower likelihood of agreement on BP control. Conversely, recent healthcare service utilization was associated with a higher likelihood of agreement in this regard. The discrepancies between self-reports and clinical measurements on hypertension were primarily attributed to FN reporting. These findings suggest the importance of integrating objective measurements into health surveys to improve data validity and to better inform public health strategies focusing on older populations. Future research is recommended to identify additional predictors of these discrepancies and establish predictive models to guide public health interventions.

## Electronic supplementary material

Below is the link to the electronic supplementary material.

Supplementary Material 1: STROBE checklist.

Supplementary Material 2: Table A1 Descriptive statistics of the study population, China, 2015.

## Data Availability

The data source of this study was a publicly available database, the China Health and Retirement Longitudinal Study, which was hosted by the National Development Center of Peking University. The data are available at http://charls.pku.edu.cn/index.htm.

## Abbreviations

CHARLS: China health and retirement longitudinal study
AOR: Adjusted odds ratios
BP: Blood pressure
BMI: Body mass index
CVD: Cardiovascular diseases
CKD: Chronic kidney disease
CI: Confidence interval
FN: False negative
FP: False positive
GDP: Gross domestic product
NCD: Noncommunicable disease
RRR: Relative-risk ratio
TN: True negative
TP: True positive
WHO: World health organization
