# Supplementary material for: Agreement between self-reported and objectively measured hypertension diagnosis and control: evidence from a nationally representative sample of community-dwelling middle‐aged and older adults in China
Source: Arch Public Health. 2024 Dec 26;82:245. doi: 10.1186/s13690-024-01456-5 (PMC11670411; doi:10.1186/s13690-024-01456-5)
Supplement: Supplementary file 2 — Supplementary Material 2: Table A1 Descriptive statistics of the study population, China, 2015. [file 13690_2024_1456_MOESM2_ESM.docx]

**Additional file 2：Table A1 Descriptive statistics** **of the study population, China, 2015**

| Variable | Total | Self-reported hypertension diagnosis | | Objectively measured hypertension diagnosis | | Agreement on hypertension diagnosis | | Self-reported hypertension control | | Objectively measured hypertension control | | Agreement on hypertension control | |
| --- | --- | --- | --- | --- | --- | --- | --- | --- | --- | --- | --- | --- | --- |
|  | N (%) | n (%) | p-value | n (%) | p-value | n (%) | p-value | n (%) | p-value | n (%) | p-value | n (%) | p-value |
| Overall | 13,071 (100.00) | 4,734 (36.22) | - | 5,838 (44.66) | - | 10,579 (80.93) |  | 3,884 (82.04) | - | 1,697 (35.85) | - | 2,113 (44.63) | - |
| ***Individual-level factor*** | | | | | | | | | | | |  |  |
| *Demographic and socioeconomic factor* | | | | | | | | | | | |  |  |
| Age (years) |  |  | 0.000 |  | 0.000 |  | 0.000 |  | 0.144 |  | 0.000 |  | 0.001 |
| Middle-aged (45-59) ^a^ | 5,768 (44.13) | 1,605 (27.83) |  | 1,971 (34.17) |  | 4,790 (83.04) |  | 1,299 (80.93) |  | 625 (39.00) |  | 768 (47.85) |  |
| Young-old (60-74) | 5,956 (45.56) | 2,492 (41.84) |  | 3,021 (50.72) |  | 4,763 (79.97) |  | 2,047 (82.14) |  | 879 (35.27) |  | 1,096 (43.98) |  |
| Old-old (75 and older) | 1,347 (10.31) | 637 (47.29) |  | 846 (62.81) |  | 1,026 (76.17) |  | 538 (84.46) |  | 192 (30.14) |  | 249 (39.09) |  |
| Sex |  |  | 0.026 |  | 0.061 |  | 0.001 |  | 0.368 |  | 0.023 |  | 0.141 |
| Female ^a^ | 6,939 (53.09) | 2,574 (37.09) |  | 3,046 (43.90) |  | 5,689 (81.99) |  | 2,100 (81.59) |  | 960 (37.30) |  | 1,174 (45.61) |  |
| Male | 6,132 (46.91) | 2,160 (35.23) |  | 2,792 (45.53) |  | 4,890 (79.75) |  | 1,784 (82.59) |  | 737 (34.12) |  | 939 (43.47) |  |
| Marriage status |  |  | 0.000 |  | 0.000 |  | 0.000 |  | 0.582 |  | 0.026 |  | 0.114 |
| Unmarried ^a^ | 1,846 (14.12) | 805 (43.61) |  | 1,035 (56.07) |  | 1,426 (77.25) |  | 655 (81.37) |  | 261 (32.42) |  | 339 (42.11) |  |
| Married | 11,225 (85.88) | 3,929 (35.00) |  | 4,803 (42.79) |  | 9,153 (81.54) |  | 3,229 (82.18) |  | 1,436 (36.55) |  | 1,774 (45.15) |  |
| Educational level |  |  | 0.000 |  | 0.000 |  | 0.031 |  | 0.001 |  | 0.104 |  | 0.606 |
| Illiteracy ^a^ | 3,519 (26.92) | 1,384 (39.33) |  | 1,693 (48.11) |  | 2,796 (79.45) |  | 1,105 (79.84) |  | 473 (34.18) |  | 618 (44.65) |  |
| Primary school | 5,424 (41.50) | 1,976 (36.43) |  | 2,394 (44.14) |  | 4,426 (81.60) |  | 1,610 (81.48) |  | 742 (37.55) |  | 896 (45.34) |  |
| Secondary school and above | 4,128 (31.58) | 1,374 (33.28) |  | 1,751 (42.42) |  | 3,357 (81.32) |  | 1,169 (85.08) |  | 482 (35.08) |  | 599 (43.60) |  |
| Occupational status |  |  | 0.000 |  | 0.000 |  | 0.797 |  | 0.174 |  | 0.000 |  | 0.001 |
| Without a job or retired ^a^ | 4,782 (36.58) | 2,174 (45.46) |  | 2,666 (55.75) |  | 3,856 (80.64) |  | 1,795 (82.57) |  | 699 (32.15) |  | 908 (41.77) |  |
| Agricultural job | 5,067 (38.77) | 1,650 (32.56) |  | 1,995 (39.37) |  | 4,112 (81.15) |  | 1,331 (80.67) |  | 646 (39.15) |  | 779 (47.21) |  |
| Non-agricultural job | 3,222 (24.65) | 910 (28.24) |  | 1,177 (36.53) |  | 2,611 (81.04) |  | 758 (83.30) |  | 352 (38.68) |  | 426 (46.81) |  |
| *Multimorbidity* | | | | | | | | | | | | | |
| Diabetes |  |  | 0.000 |  | 0.000 |  | 0.589 |  | 0.083 |  | 0.000 |  | 0.000 |
| Without ^a^ | 11,813 (90.38) | 3,949 (33.43) |  | 4,944 (41.85) |  | 9,568 (81.00) |  | 3,257 (82.48) |  | 1,479 (37.45) |  | 1,811 (45.86) |  |
| With | 1,258 (9.62) | 785 (62.40) |  | 894 (71.07) |  | 1,011 (80.37) |  | 627 (79.87) |  | 218 (27.77) |  | 302 (38.47) |  |
| CVD |  |  | 0.000 |  | 0.000 |  | 0.251 |  | 0.020 |  | 0.000 |  | 0.000 |
| Without ^a^ | 9,000 (68.85) | 2,405 (26.72) |  | 3,109 (34.54) |  | 7,308 (81.20) |  | 2,004 (83.33) |  | 1,080 (44.91) |  | 1,213 (50.44) |  |
| With | 4,071 (31.15) | 2,329 (57.21) |  | 2,729 (67.04) |  | 3,271 (80.35) |  | 1,880 (80.72) |  | 617 (26.49) |  | 900 (38.64) |  |
| CKD |  |  | 0.000 |  | 0.000 |  | 0.018 |  | 0.258 |  | 0.002 |  | 0.041 |
| Without ^a^ | 11,804 (90.31) | 4,175 (35.37) |  | 5,211 (44.15) |  | 9,522 (80.67) |  | 3,435 (82.28) |  | 1,530 (36.65) |  | 1,886 (45.17) |  |
| With | 1,267 (9.69) | 559 (44.12) |  | 627 (49.49) |  | 1,057 (83.43) |  | 449 (80.32) |  | 167 (29.87) |  | 227 (40.61) |  |
| *Behavioural risk* |  |  |  |  |  |  |  |  |  |  |  |  |  |
| Smoking |  |  | 0.000 |  | 0.000 |  | 0.049 |  | 0.213 |  | 0.254 |  | 0.625 |
| Never ^a^ | 7,310 (55.93) | 2,680 (36.66) |  | 3,192 (43.67) |  | 5,970 (81.67) |  | 2,185 (81.53) |  | 983 (36.70) |  | 1,212 (45.22) |  |
| Smoking quitter | 2,149 (16.44) | 921 (42.86) |  | 1,128 (52.49) |  | 1,712 (79.66) |  | 774 (84.04) |  | 310 (33.66) |  | 401 (43.54) |  |
| Current Smoker | 3,612 (27.63) | 1,133 (31.37) |  | 1,518 (42.03) |  | 2,897 (80.20) |  | 925 (81.64) |  | 404 (35.66) |  | 500 (44.13) |  |
| Heavy drinking |  |  | 0.089 |  | 0.011 |  | 0.000 |  | 0.373 |  | 0.015 |  | 0.007 |
| No ^a^ | 11,163 (85.40) | 4,076 (36.51) |  | 4,935 (44.21) |  | 9,132 (81.81) |  | 3,336 (81.84) |  | 1,489 (36.53) |  | 1,851 (45.41) |  |
| Yes | 1,908 (14.60) | 658 (34.49) |  | 903 (47.33) |  | 1,447 (75.84) |  | 548 (83.28) |  | 208 (31.61) |  | 262 (39.82) |  |
| Overweight/obesity |  |  | 0.000 |  | 0.000 |  | 0.486 |  | 0.010 |  | 0.000 |  | 0.000 |
| No ^a^ | 8,408 (64.33) | 2,411 (28.68) |  | 3,127 (37.19) |  | 6,820 (81.11) |  | 1,944 (80.63) |  | 946 (39.24) |  | 1,153 (47.82) |  |
| Yes | 4,663 (35.67) | 2,323 (49.82) |  | 2,711 (58.14) |  | 3,759 (80.61) |  | 1,940 (83.51) |  | 751 (32.33) |  | 960 (41.33) |  |
| *Healthcare-seeking behaviour* | | | | | | | | | | | | | |
| Routine physical examination |  |  | 0.000 |  | 0.000 |  | 0.206 |  | 0.005 |  | 0.109 |  | 0.013 |
| No ^a^ | 7,595 (58.11) | 2,342 (30.84) |  | 3,036 (39.97) |  | 6,119 (80.57) |  | 1,884 (80.44) |  | 866 (36.98) |  | 1,088 (46.46) |  |
| Yes | 5,476 (41.89) | 2,392 (43.68) |  | 2,802 (51.17) |  | 4,460 (81.45) |  | 2,000 (83.61) |  | 831 (34.74) |  | 1,025 (42.85) |  |
| Hospital admission |  |  | 0.000 |  | 0.000 |  | 0.003 |  | 0.000 |  | 0.062 |  | 0.023 |
| No ^a^ | 11,185 (85.57) | 3,841 (34.34) |  | 4,825 (43.14) |  | 9,005 (80.51) |  | 3,202 (83.36) |  | 1,401 (36.47) |  | 1,684 (43.84) |  |
| Yes | 1,886 (14.43) | 893 (47.35) |  | 1,013 (53.71) |  | 1,574 (83.46) |  | 682 (76.37) |  | 296 (33.15) |  | 429 (48.04) |  |
| Outpatient care |  |  | 0.000 |  | 0.004 |  | 0.000 |  | 0.000 |  | 0.994 |  | 0.056 |
| No ^a^ | 10,568 (80.85) | 3,677 (34.79) |  | 4,655 (44.05) |  | 8,472 (80.17) |  | 3,057 (83.14) |  | 1,318 (35.84) |  | 1,614 (43.89) |  |
| Yes | 2,503 (19.15) | 1,057 (42.23) |  | 1,183 (47.26) |  | 2,107 (84.18) |  | 827 (78.24) |  | 379 (35.86) |  | 499 (47.21) |  |
| Antihypertensive medication adherence | |  | 0.000 |  | 0.000 |  | 0.000 |  | 0.000 |  | 0.000 |  | 0.000 |
| No ^a^ | 9,833 (75.23) | 1,496 (15.21) |  | 2,600 (26.44) |  | 7,341 (74.66) |  | 1,274 (85.16) |  | 680 (45.45) |  | 748 (50.00) |  |
| Yes | 3,238 (24.77) | 3,238 (100.00) |  | 3,238 (100.00) |  | 3,238 (100.00) |  | 2,610 (80.61) |  | 1,017 (31.41) |  | 1,365 (42.16) |  |
| Regular BP monitoring | |  | 0.000 |  | 0.000 |  | 0.000 |  | 0.948 |  | 0.000 |  | 0.016 |
| No ^a^ | 12,606 (96.44) | 4,269 (33.86) |  | 5,387 (42.73) |  | 10,128 (80.34) |  | 4,269 (82.06) |  | 1,566 (36.68) |  | 1,930 (45.21) |  |
| Yes | 465 (3.56) | 465 (100.00) |  | 451 (96.99) |  | 451 (96.99) |  | 465 (81.94) |  | 131 (28.17) |  | 183 (39.35) |  |
| ***Household-level factor*** | |  |  |  |  |  |  |  |  |  |  |  |  |
| Household economic level (quintile) | |  | 0.003 |  | 0.332 |  | 0.110 |  | 0.021 |  | 0.858 |  | 0.958 |
| 1st (lowest) ^a^ | 2,618 (20.03) | 894 (34.15) |  | 1,136 (43.39) |  | 2,076 (79.30) |  | 752 (84.12) |  | 330 (36.91) |  | 408 (45.64) |  |
| 2nd | 2,627 (20.10) | 924 (35.17) |  | 1,167 (44.42) |  | 2,116 (80.55) |  | 738 (79.87) |  | 327 (35.39) |  | 409 (44.26) |  |
| 3rd | 2,599 (19.88) | 921 (35.44) |  | 1,153 (44.36) |  | 2,115 (81.38) |  | 743 (80.67) |  | 319 (34.64) |  | 413 (44.84) |  |
| 4th | 2,636 (20.17) | 1,018 (38.62) |  | 1,219 (46.24) |  | 2,147 (81.45) |  | 825 (81.04) |  | 364 (35.76) |  | 447 (43.91) |  |
| 5th (highest) | 2,591 (19.82) | 977 (37.71) |  | 1,163 (44.89) |  | 2,125 (82.01) |  | 826 (84.54) |  | 357 (36.54) |  | 436 (44.63) |  |
| ***Community-level factor*** |  |  |  |  |  |  |  |  |  |  |  |  |  |
| Area of residence |  |  | 0.000 |  | 0.000 |  | 0.224 |  | 0.000 |  | 0.002 |  | 0.000 |
| Urban ^a^ | 4,722 (36.13) | 1,806 (38.25) |  | 2,276 (48.20) |  | 3,848 (81.49) |  | 1,542 (85.38) |  | 598 (33.11) |  | 748 (41.42) |  |
| Rural | 8,349 (63.87) | 2,928 (35.07) |  | 3,562 (42.66) |  | 6,731 (80.62) |  | 2,342 (79.99) |  | 1,099 (37.51) |  | 1,365 (46.62) |  |
| Local economic level |  |  | 0.043 |  | 0.005 |  | 0.659 |  | 0.000 |  | 0.983 |  | 0.065 |
| Underdeveloped ^a^ | 8,285 (63.38) | 2,947 (35.57) |  | 3,623 (43.73) |  | 6,715 (81.05) |  | 2,348 (79.67) |  | 1,053 (35.80) |  | 1,346 (45.67) |  |
| Developed | 4,786 (36.62) | 1,787 (37.34) |  | 2,215 (46.28) |  | 3,864 (80.74) |  | 1,536 (85.95) |  | 640 (35.53) |  | 767 (42.92) |  |

Source: China Health and Retirement Longitudinal Study (CHARLS), 2015.

Note: ^a^ Reference group. Results are presented as number (proportion).
